# A Histoplasma capsulatum Lipid Metabolic Map Identifies Antifungal Targets

**DOI:** 10.1128/mBio.02972-21

**Published:** 2021-11-23

**Authors:** Daniel Zamith-Miranda, Heino M. Heyman, Meagan C. Burnet, Sneha P. Couvillion, Xueyun Zheng, Nathalie Munoz, William C. Nelson, Jennifer E. Kyle, Erika M. Zink, Karl K. Weitz, Kent J. Bloodsworth, Geremy Clair, Jeremy D. Zucker, Jeremy R. Teuton, Samuel H. Payne, Young-Mo Kim, Morayma Reyes Gil, Erin S. Baker, Erin L. Bredeweg, Joshua D. Nosanchuk, Ernesto S. Nakayasu

**Affiliations:** a Department of Microbiology and Immunology, Albert Einstein College of Medicine, Bronx, New York, USA; b Division of Infectious Diseases, Department of Medicine, Albert Einstein College of Medicine, Bronx, New York, USA; c Biological Sciences Division, Pacific Northwest National Laboratory, Richland, Washington, USA; d Environmental and Molecular Sciences Laboratory, Pacific Northwest National Laboratory, Richland, Washington, USA; e Department of Biology, Brigham Young University, Provo, Utah, USA; f Hematology Laboratory, Department of Pathology, Albert Einstein College of Medicine, Bronx, New York, USA; g Department of Chemistry, North Carolina State University, Raleigh, North Carolina, USA; University of British Columbia

**Keywords:** *Histoplasma capsulatum*, drug targets, lipid metabolism, lipidomics, metabolic map, proteomics

## Abstract

Lipids play a fundamental role in fungal cell biology, being essential cell membrane components and major targets of antifungal drugs. A deeper knowledge of lipid metabolism is key for developing new drugs and a better understanding of fungal pathogenesis. Here, we built a comprehensive map of the Histoplasma capsulatum lipid metabolic pathway by incorporating proteomic and lipidomic analyses. We performed genetic complementation and overexpression of H. capsulatum genes in Saccharomyces cerevisiae to validate reactions identified in the map and to determine enzymes responsible for catalyzing orphan reactions. The map led to the identification of both the fatty acid desaturation and the sphingolipid biosynthesis pathways as targets for drug development. We found that the sphingolipid biosynthesis inhibitor myriocin, the fatty acid desaturase inhibitor thiocarlide, and the fatty acid analog 10-thiastearic acid inhibit H. capsulatum growth in nanomolar to low-micromolar concentrations. These compounds also reduced the intracellular infection in an alveolar macrophage cell line. Overall, this lipid metabolic map revealed pathways that can be targeted for drug development.

## INTRODUCTION

Fungal diseases affect more than 1 billion people and cause 1.6 million deaths every year (1). Histoplasma capsulatum, the causative agent of histoplasmosis, is an important pathogen in this context, being associated with HIV infections and causing morbidity and mortality worldwide (2). Data from 2011 to 2014 identified 3,409 cases of histoplasmosis in 12 states in the United States with a 7% mortality rate (3). Serological surveys have shown reactivity against the H. capsulatum antigen histoplasmin in 60 to 90% of individuals in communities surrounding the Mississippi and Ohio basins, suggesting that the epidemiological data are underestimated (4, 5). Histoplasmosis treatment relies on only a few antifungals, and the increasing number of resistant strains is a major public health concern (6, 7). The absence of immunotherapies and the fact that the newest class of antifungal drugs, echinocandins, are ineffective against H. capsulatum make the development of new therapies a high priority. A major hurdle in developing new drugs is the limited knowledge about the detailed metabolic reactions of this microbe.

Lipids have essential roles in many biological processes, and the biosynthetic pathways of fungal lipids diverged from metazoan pathways, which makes them obvious antifungal drug targets (8). Indeed, two classes of current antifungal drugs target lipid metabolism: (i) polyenes, including amphotericin B and nystatin, bind and extract ergosterol from the fungal membrane (9–11); and (ii) azoles, such as voriconazole, itraconazole, and fluconazole, inhibit cytochrome P-450-dependent 14α-sterol demethylase, an enzyme of the ergosterol biosynthetic pathway (8, 12). More recently, glucosylceramide, a type of sphingolipid that is critical for infection of many fungal species, has been validated as a drug target in Cryptococcus neoformans, Aspergillus fumigatus, Candida auris, and *Sporothrix* spp. (13–17).

There is limited information about H. capsulatum lipid composition and function, which is also true for most pathogenic fungi. Polyenes and azoles have been used to treat most mycoses, but many of these fungal species might not even produce ergosterol. Some species produce cholesterol, brassicasterol, lanosterol, and other sterols as final products (18–22). However, it is still unclear how the different sterols affect the efficacy of polyenes and azoles. Considering the importance of lipids, we reasoned that a deeper characterization of H. capsulatum lipid metabolism would result in the discovery of drug targets. In this study, we performed an in-depth characterization of the H. capsulatum lipid biosynthetic pathway by profiling its lipids and the associated proteins, which were incorporated into a metabolic map. By comparing our results to those for Saccharomyces cerevisiae and humans, we show unique features of the lipid biosynthetic pathway of H. capsulatum that can be targeted for drug development.

## RESULTS

### Proteomic and lipidomic analyses and overview of the H. capsulatum lipid metabolism.

**FIG 1:**
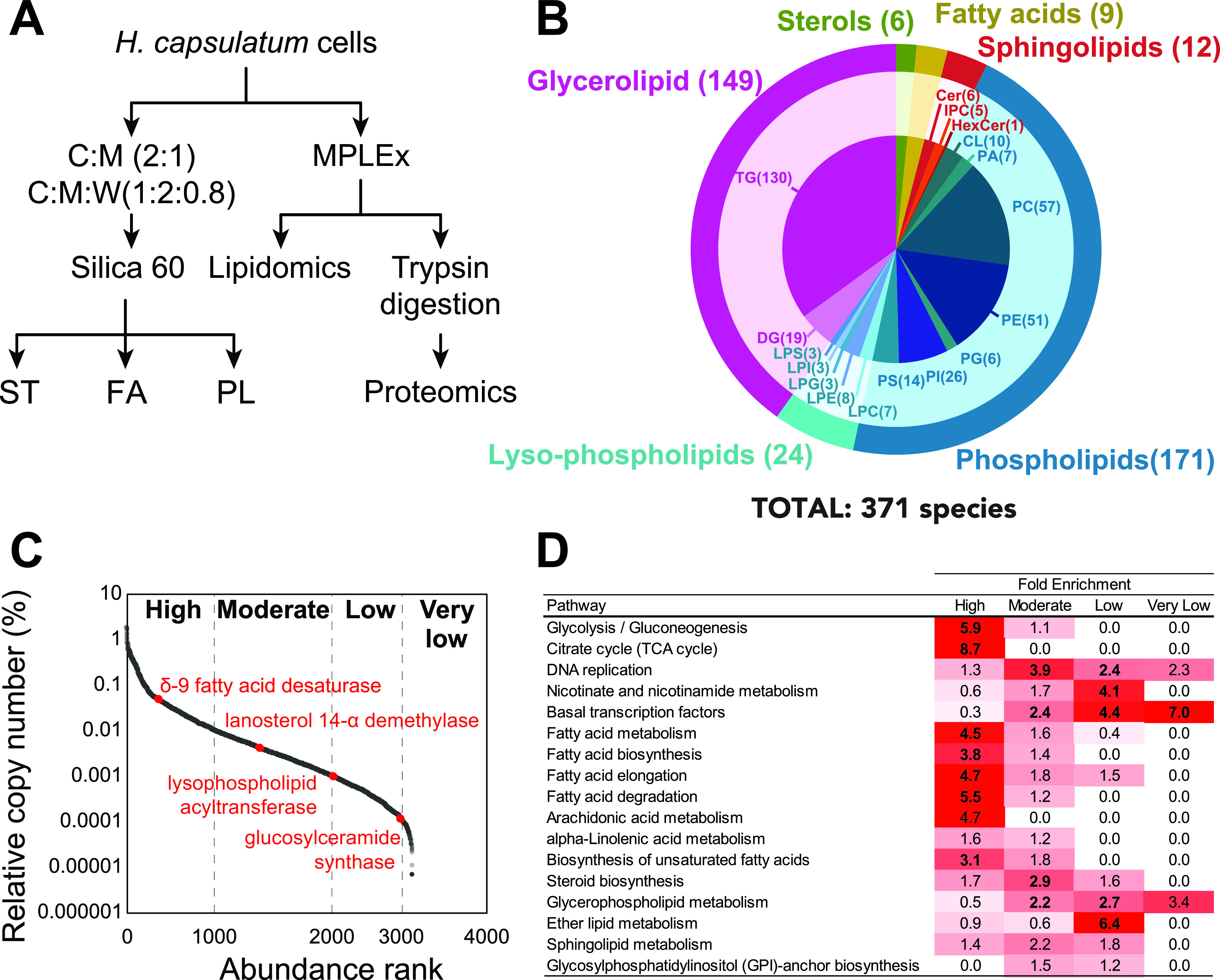
Proteomic and lipidomic analyses of Histoplasma capsulatum yeast cells. (A) Extraction procedure for lipidomic proteomics analyses. Yeast cells were extracted sequentially with chloroform-methanol (2:1, vol/vol) and chloroform-methanol-water (1:2:0.8, vol/vol/vol) and fractionated by Silica 60 solid-phase extraction for sterol, free fatty acid, and phospholipid analyses. Cells were also submitted to simultaneous metabolite, protein, and lipid extraction (MPLEx) for global lipidomics and proteomics analyses. (B) Overall lipid coverage combining MPLEx and specific lipid extractions of Histoplasma capsulatum. (C) Protein abundance classification based on their relative copy numbers. (D) Function enrichment analysis based on the KEGG annotation of H. capsulatum proteins with different abundance levels. Abbreviations: C, chloroform; Cer, ceramide; CL, cardiolipin; DG, diacylglycerol; FA, fatty acid; HexCer, hexosylceramide; LPC, lysophosphatidylcholine; LPE, lysophosphatidylethanolamines; LPG, lysophosphatidylglycerol; LPI, lysophosphatidylinositol; LPS, lysophosphatidylserine; M, methanol; PA, phosphatidic acid; PC, phosphatidylinositol; PE, phosphatidylethanolamine; PG, phosphatidylglycerol; PI, phosphatidylinositol; PI_Cer, inositolphosphoceramide; PL, phospholipid; PS, phosphatidylserine; ST, sterol; TG, triacylglycerol; W, water.

To determine the global landscape of the lipid metabolic pathway, we performed comprehensive lipidomic and proteomic analyses of log-phase yeast-form H. capsulatum grown in Ham’s F12 medium. We chose this medium since it is a defined medium, allowing us to distinguish lipids that are synthesized by the fungus versus the ones obtained from nutrients. Total lipids were extracted using two different methods and submitted to chromatographic separation before analyses by mass spectrometry. Paired global lipidomics and proteomics analyses were performed by submitting the samples to a simultaneous metabolite, protein and lipid extraction (MPLEx) (23) (Fig. 1A). For complementary analyses of sterols, free fatty acids, and phospholipids, yeast cells were extracted with two rounds of organic solvent extraction, followed by a solid-phase fractionation in a silica 60 column (Fig. 1A). The extracted fractions were analyzed by either gas chromatography-mass spectrometry (GC-MS) (sterols and fatty acids) or liquid chromatography-tandem mass spectrometry (LC-MS/MS) (sphingolipids, glycerolipids, phospholipids, and proteins) (Fig. 1A).

The combined analysis identified 371 unique lipid species from 5 major lipid categories (fatty acids, sterols, glycerolipids, sphingolipids, and glycerophospholipids [phospholipids and lysophospholipids]) that were subdivided into 19 subclasses (Fig. 1B) (see Table S1 to S8 at https://osf.io/ku8ta/). The most diverse subclasses of lipids in terms of the number of identified species were triacylglycerols (TG), phosphatidylcholines (PC), and phosphatidylethanolamine (PE), with 130, 57, and 51 individual species, respectively (Fig. 1B). The proteomic analysis led to the identification of 3,215 proteins (see Table S9 at https://osf.io/ku8ta/). To provide a measurement of the protein abundances in the cells, we calculated the relative copy number of each protein and scaled them into high, moderate, low, and very low abundance (Fig. 1C), using a scale similar to one previously described (24). To validate this scale, we performed a function enrichment analysis using the KEGG annotation to check the abundance of different pathways. As expected, glycolysis/gluconeogenesis and tricarboxylic acid (TCA) cycle were overrepresented among the highly abundant proteins, whereas DNA replication, nicotinate and nicotinamide metabolism, and basal transcription factors were enriched in moderate, low, and very low abundance levels, respectively (Fig. 1D). The same scale showed that fatty acid metabolism was enriched in highly abundant proteins, whereas steroid biosynthesis proteins were mainly present in moderate abundance (Fig. 1C and D). Glycerophospholipid and sphingolipid metabolism proteins were not concentrated in a single abundance level and were spread mostly between moderate- and low-abundance levels (Fig. 1C and D). The abundance levels of the proteins were directly proportional to each type of lipid in the cellular membrane. For instance, the proteins of fatty acid and steroid (the building blocks of the cell membrane; therefore, most abundant lipids) metabolism were significantly enriched among the high- and moderate-abundance proteins (highlighted in boldface in the first two columns of Fig. 1D). Metabolisms of glycerophospholipid, ether lipid, and sphingolipid, which are responsible for the synthesis of lipids from specific lower-abundance classes, were enriched in proteins with moderate to very low abundance proteins (Fig. 1D).

### Lipid biosynthesis and remodeling map of H. capsulatum.

**FIG 2:**
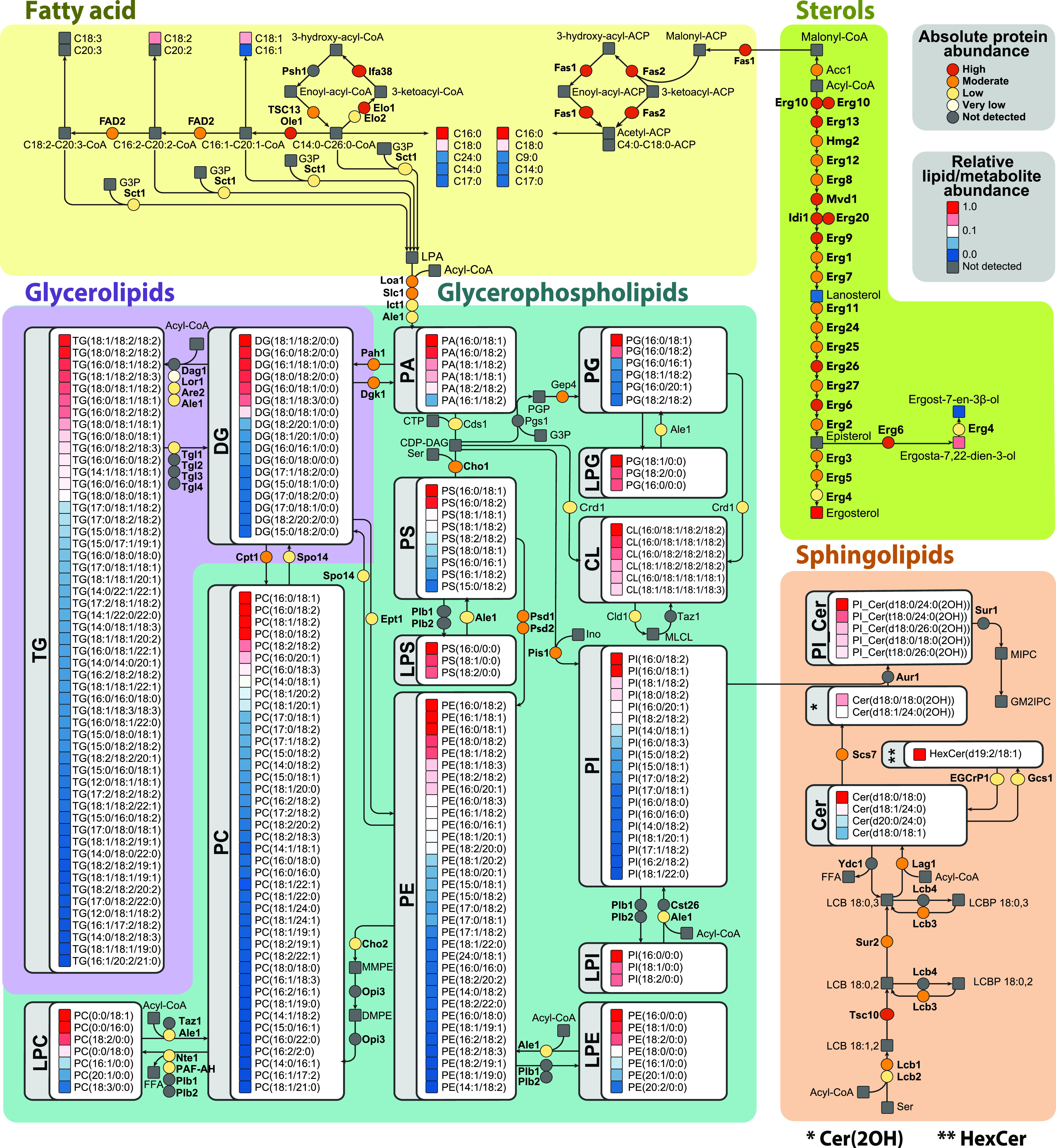
Metabolic map of Histoplasma capsulatum lipids and biosynthesis proteins. The map was built based on genomic information along with proteomics and lipidomics data. Lipid abundances were normalized by the most intense signal in the mass spectrometry analysis within each lipid class, whereas the proteins were quantified based on their relative copy number. For details about enzyme names and homologs, see Table S10 at https://osf.io/ku8ta/. Abbreviations: Cer, ceramide; CL, cardiolipin; DG, diacylglycerol; HexCer, hexosylceramide; LPC, lysophosphatidylcholine; LPE, lysophosphatidylethanolamines; LPG, lysophosphatidylglycerol; LPI, lysophosphatidylinositol; LPS, lysophosphatidylserine; M, methanol; PA, phosphatidic acid; PC, phosphatidylinositol; PE, phosphatidylethanolamine; PG, phosphatidylglycerol; PI, phosphatidylinositol; PI_Cer, inositolphosphoceramide; PL, phospholipid; PS, phosphatidylserine; TG, triacylglycerol.

To provide a global view of H. capsulatum lipid metabolism, we built a map including lipid biosynthesis and remodeling reactions. The map was constructed based on a metabolic model (25) structured according to conserved metabolism between H. capsulatum and S. cerevisiae. We added information available for Cryptococcus neoformans, one of the best-characterized fungal organisms in terms of lipid metabolism (see Table S10 at https://osf.io/ku8ta/), along with previous literature on H. capsulatum. We used the H. capsulatum genomic, proteomic, and lipidomic information to further restrict or add reactions that are present in this organism. The H. capsulatum map was incorporated with the relative abundance of the lipid species within the same subclass and the protein abundances (Fig. 2). In terms of fatty acids, species containing 16 and 18 carbons were most abundant. Consistent with Fig. 1D, 5 out of 10 proteins of this pathway were highly abundant (Fig. 2). Similarly, 13 of the 23 sterol biosynthesis proteins had moderate abundance, with ergosterol being the most abundant product (Fig. 2). In the sphingolipid pathway, ceramides (Cer), hexosylceramide (HexCer), and inositolphosphoceramides (PI_Cer) were the detected lipid species (Fig. 2). Out of the 9 proteins detected in the proteome, 3 had low abundance, 5 had moderate abundance, and 1 was highly abundant (Fig. 2). The number of low-abundance proteins does not necessarily mean that the pathway has low activity. For instance, one low-abundance protein had more abundant paralogues with the same function (serine palmitoyltransferase Lcb2 versus Lcb1), and the other two proteins regulate the specific modification of the head group of HexCer, glucosylceramide synthase Gcs1 and endoglycoceramidase-related protein EGCrP1 (Fig. 2). In terms of glycerolipids, diacylglycerols (DGs) and TGs were identified, being the proteins from this pathway that were present at moderate (2), low (3), and very low abundance (1) (Fig. 2). Like the free fatty acid composition, the most abundant species of DGs and TGs had fatty acyl groups with either 16 or 18 carbons attached to them (Fig. 2). Consistent with the glycerolipids, all the different glycerophospholipid classes had species bearing C_16_ and C_18_ as the most abundant in each (Fig. 2). To increase the utility of the lipid metabolic map, we developed the map into an informatics tool to visualize lipidomics and proteomics data. We tested the tool using lipidomics and proteomics data of Candida albicans from a previous publication (26), which showed a similar pattern of lipid species, with fatty acids containing 16 or 18 carbons being the most abundant ones (see Fig. S1 at https://osf.io/ku8ta/).

**FIG 3:**
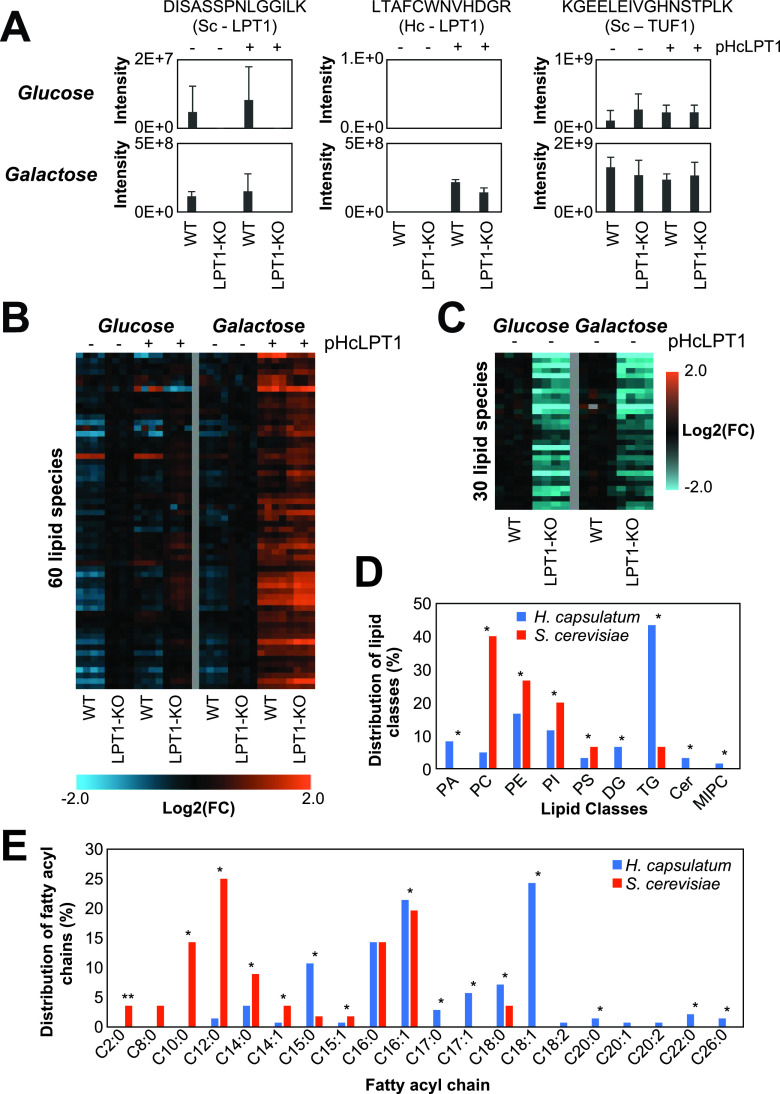
Analysis of Saccharomyces cerevisiae (Sc) and Histoplasma capsulatum (Hc) lysophospholipid acetyltransferase LPT1 genes. The analysis was done in wild-type and LPT1-knockout (KO) (*Lpt1*^−/−^) S. cerevisiae strains complemented or not with the pESC-URA plasmid with the Hc-LPT1 gene under a galactose-inducible promoter. S. cerevisiae WT and LPT1-knockout (KO) strains were grown in YNB medium supplemented with glucose (Glc) or galactose (Gal). (A) Protein abundance of Sc and Hc LPT1 proteins measured by the intensity of specific peptides in the LC-MS/MS analysis. Elongation factor Tu (TUF1) was used as a loading control. As expected, Sc LPT1 was only detected in the WT strain, whereas Hc LPT1 was detected only in strains transformed with the plasmid and induced with galactose. (B and C) Heatmaps of identified HcLPT1 (B) and ScLPT1 (C) products (*P* ≤ 0.05) compared to the complemented versus control strains (B) or LPT1-KO versus WT strains (C). The experiments were done in two independent batches delimited by the vertical gray lines. (D and E) Distribution of the lipid classes (D) and fatty acyl groups (E) of the HcLPT1 and ScLPT1 products. *, *P* ≤ 0.05 by Fisher’s exact test.

We further curated the map because homologous genes can have different specificities. We chose to determine the substrates and products of the H. capsulatum acyltransferase Ale1 (also known as lysophosphatidylcholine [LPC] acyltransferase 1 [LPT1]), because its homolog in S. cerevisiae has been shown to modify different classes of lipids (27). This would allow coverage of a large portion of H. capsulatum lipid metabolism. Unfortunately, gene knockout is very difficult in H. capsulatum, and RNA interference experiments often result in a reduction of expression levels that fail to produce a phenotype. Conversely, exogenous expression of genes from the lipid metabolism in biotechnological fungi, such as S. cerevisiae and Yarrowia lipolytica, has provided important insights on enzyme specificity (28, 29). Therefore, we performed complementation studies in S. cerevisiae with H. capsulatum *Lpt1* (*Hc-Lpt1*), which would allow curation of a large portion of the metabolic map. A lipidomic analysis was performed in the *Lpt1* knockout strain of S. cerevisiae complemented or not with the *Hc-Lpt1* homolog (53% similarity to S. cerevisiae *Lpt1*; *Sc-Lpt1*) (see Fig. S2 at https://osf.io/ku8ta/), using a plasmid with a galactose-inducible promoter. This allows specific expression of *Hc-Lpt1* in the presence of galactose but not glucose. Both gene knockout and recombinant expression were validated by proteomic analysis (Fig. 3A). As expected, the Sc-LPT1 specific peptide DISASSPNLGGILK was detected in wild-type S. cerevisiae in both glucose- and galactose-supplemented media (Fig. 3A). The Hc-LPT1-specific peptide LTAFCWNVHDGR was detected only in complemented strains that were grown in galactose-supplemented medium (Fig. 3A). The abundance of the peptide KGEELEIVGHNSTPLK from the housekeeping protein elongation factor Tu (TUF1) was similar across different samples (Fig. 3A).

The lipidomics analysis showed that Hc-LPT1 complementation increased the abundances of 60 lipids, including phosphatidic acid (PA), PC, PE, phosphatidylinositol (PI), PS, DG, TG, Cer, and mannosyl-inositolphosphoceramide (MIPC) (Fig. 3B to D) (see Table S11 at https://osf.io/ku8ta/). The Hc-LPT1 complementation also significantly reduced the levels of 77 lipids, including 5 LPC, 3 lysophosphatidylethanolamines (LPE), 2 sphinganines, and 3 DGs (see Table S12 at https://osf.io/ku8ta/). On the other hand, disruption of the Sc-LPT1 gene reduced the levels of 30 potential products, including PC, PE, PI, PS, and TG. Notably, the proportion of acylated products between LPT1s from the two species was very different, with PC, PE, PI, and PS being more acylated by the S. cerevisiae homolog and PA, DG, TG, Cer, and MIPC by the H. capsulatum counterpart (Fig. 3D) (see Table S13 at https://osf.io/ku8ta/). Sc-LPT1 showed a preference for production of lipid species containing short fatty acyl chains, with all the identified products having chains with ≤14 carbons (Fig. 3E). Complementation with the *Hc-Lpt1* gene showed a different phenotype, as this homolog produced lipid species containing odd carbon numbers (C_15_ and C_17_) and longer (≥C_18_) fatty acyl chains (Fig. 3E). Overall, the map provides a global view of lipids being produced by H. capsulatum along with catalytic enzymes.

### Evolutionarily divergent lipid metabolic pathways as drug targets.

**FIG 4:**
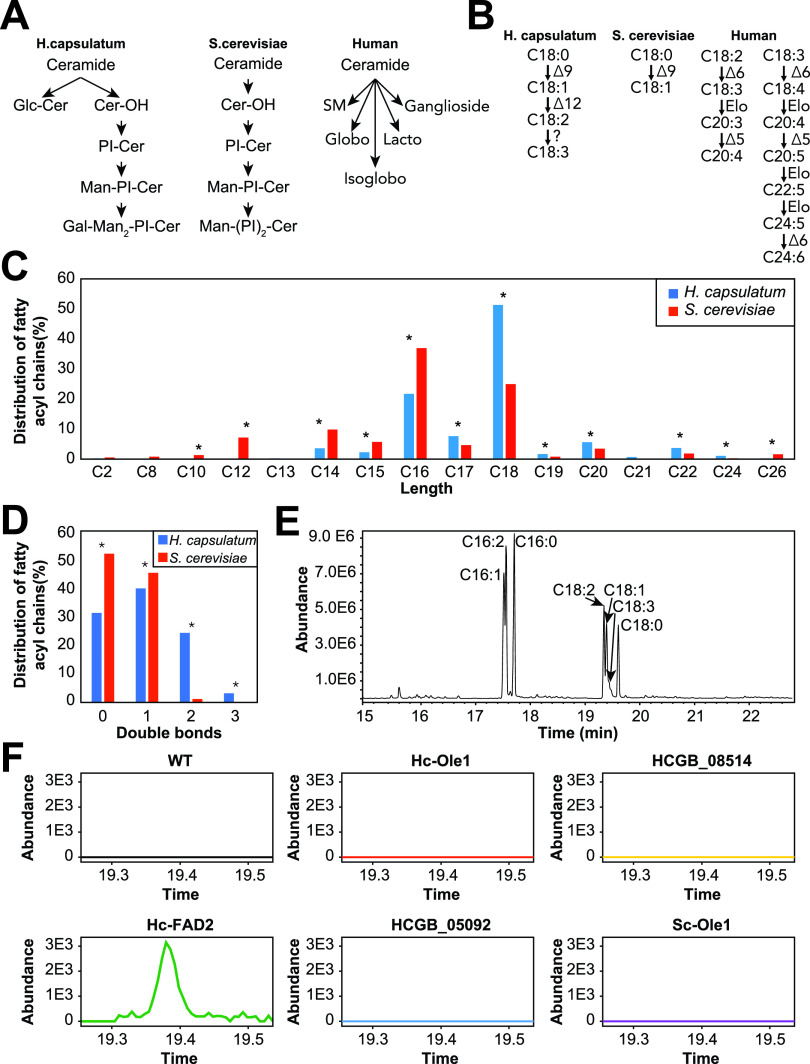
Sphingolipid and fatty acid metabolism in Histoplasma capsulatum. (A) Sphingolipids of H. capsulatum, Saccharomyces cerevisiae, and humans. (B) Fatty acid desaturase pathways in H. capsulatum, S. cerevisiae, and humans. (C) Distribution of carbon chain lengths of fatty acyl groups from lipids in global lipidomic analysis. (D) Distribution of double bonds on fatty acyl groups from lipids in global lipidomic analysis. (E) Representative chromatogram of fatty acid analysis from S. cerevisiae cells expressing the H. capsulatum FAD2 gene. The analysis was done in an S. cerevisiae strain transformed with the pESC-URA plasmid with the Hc-FAD2 gene under a galactose-inducible promoter. Cells were grown in YNB medium supplemented with galactose. The chromatogram shows the production of fatty acids with 2 and 3 double bonds. (F) Extracted ion chromatogram of α-linolenic acid (C_18:3_; 9Z, 12Z, 15Z) of S. cerevisiae cells expressing various fatty acid desaturase candidates. α-Linolenic acid was only detected in cells expressing the H. capsulatum FAD2 gene. *, *P* ≤ 0.05 by Fisher’s exact test.

We next focused on pathways that could be targeted for antifungal chemotherapies. The ideal drug target is essential for the fungal survival and divergence (substantially different or absent) compared to humans to allow specificity and reduce the chances of side effects. Therefore, we searched the lipid metabolic map for lipid pathways with enzymes that were present in H. capsulatum but had no homologs in humans or S. cerevisiae as potential candidates for anti-H. capsulatum drug targets. The most divergent pathways were sphingolipid and fatty acid metabolism (Fig. 4A and B). S. cerevisiae mainly produces (glyco)inositolphosphoceramides and humans produce complex glycosphingolipids, whereas H. capsulatum produces both (glyco)inositolphosphoceramides and simpler glycosphingolipids (Fig. 4A).

In fatty acid metabolism, humans are unable to perform the first two steps of fatty acid desaturation and instead take up the respective unsaturated fatty acids from food, while S. cerevisiae has only one fatty acid desaturase gene (delta-9 desaturase, also known as oleate synthase *Ole1*) in its genome (Fig. 4B). In addition to Ole1, H. capsulatum has a gene annotated as delta-12 desaturase (*FAD2*) and two uncharacterized desaturases (HCBG_08514 and HCBG_05092), which collectively might be responsible for the production of fatty acids with 2 and 3 double bonds (Fig. 4B). We examined the distribution of fatty acyl chains that are incorporated into H. capsulatum and S. cerevisiae lipids. The results showed that both fungi incorporate fatty acyl groups with different chain lengths into their lipids. S. cerevisiae incorporates significantly more fatty acyl chains with fewer than 16 carbons (by Fisher’s exact test), whereas H. capsulatum has significantly more lipids with fatty acids longer than 17 carbons (Fig. 4C). In terms of unsaturation, S. cerevisiae had almost exclusively saturated lipids or fatty acyl chains with 1 double bond, whereas H. capsulatum had fatty acyl chains with 2 or 3 double bonds (Fig. 4D). To develop further insights on the enzymes responsible for desaturating these fatty acids, we expressed all 4 H. capsulatum desaturase candidates in S. cerevisiae by galactose induction and determined the fatty acid composition by methylating them and analyzing them by GC-MS. We also included S. cerevisiae Ole1 as a positive control for overexpression. S. cerevisiae Ole1 overexpression increased the ratios of C_14:1_/C_14:0_, C_16:1_/C_16:0_, and C_18:1_/C_18:0_ by 4.0-, 3.0-, and 9.4-fold, respectively (see Fig. S3A at https://osf.io/ku8ta/), whereas H. capsulatum Ole1 increased the ratios of C_14:1_/C_14:0_, C_16:1_/C_16:0_, and C_18:1_/C_18:0_ by 11%, 24%, and 81%, respectively (see Fig. S3A at https://osf.io/ku8ta/). The expression of HCBG_08514 and HCBG_05092 had almost no impact on the saturation levels of the fatty acids (see Fig. S3A at https://osf.io/ku8ta/), which could be due to the lack of the precursors in S. cerevisiae. C_16:2_ (9Z,12Z-hexadecadienoic acid), C_18:2_ (9Z,12Z-linoleic acid), and C_18:3_ (9Z,12Z,15Z-α-linolenic acid) were only detected when H. capsulatum FAD2 was expressed (see Fig. S3A and B at https://osf.io/ku8ta/). A composition analysis of H. capsulatum fatty acids revealed two low-abundance peaks of C_18:3_, one being α-linolenic acid and a second peak not matching any available standard (Fig. 4E and F) (see Fig. S3C at https://osf.io/ku8ta/). These results showed that H. capsulatum FAD2 is indeed a bifunctional delta-12/delta-15 desaturase, which helped to further curate the lipid metabolic map (Fig. 2, top left). Similar to sphingolipids, the H. capsulatum fatty acid desaturase pathway represents a potential target.

**FIG 5:**
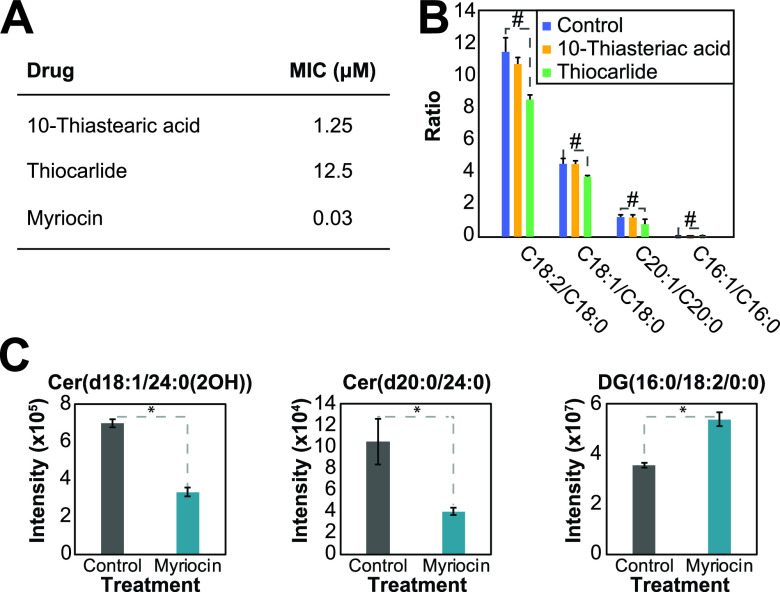
Targeting sphingolipid and fatty acid desaturation pathways for drug development. (A) MICs of 10-thiastearic acid, thiocarlide, and myriocin. The MICs were obtained from 5 independent experiments with 4 replicates each. (B) Effect of fatty acid desaturase inhibitors on the fatty acid profile. Cells were grown in half of each compound MIC and submitted to fatty acid analysis. The values represent the ratio of peak areas of unsaturated to saturated fatty acids. (C) Effect of myriocin on the lipidomics profile. Cells were grown in half the myriocin MIC and submitted to fatty acid analysis. The values represent the mass spectral signal intensity on the apex of the peak. *, *P* ≤ 0.05 by Student's *t* test when comparing the control and treatments.

To perform a proof of concept that sphingolipid and fatty acid desaturation pathways can be targeted for drug development, we tested the effects of myriocin (serine-palmitoyltransferase inhibitor, which blocks the first step of the sphingolipid biosynthesis pathway), 10-thiastearic acid, and thiocarlide (two fatty acid desaturase inhibitors) on H. capsulatum growth. Myriocin, 10-thiastearic acid, and thiocarlide showed MICs of 0.03, 1.25, and 12.5 μM, respectively (Fig. 5A). To confirm that these compounds target fatty acid desaturases and sphingolipid biosynthesis, we performed a fatty acid analysis on treated and unexposed yeast cells. Cells were grown for 48 h with half the MIC, and fatty acids were extracted, methylated, and analyzed by GC-MS. 10-Thiastearic acid did not affect the ratio between unsaturated and saturated fatty acids (Fig. 5B), suggesting that this compound targets other pathways in H. capsulatum rather than fatty acid desaturation. As expected, thiocarlide reduced the C_18:2_/C_18:0_ peak area ratio from 11.4 to 8.5 (25% reduction), the C_18:1_/C_18:0_ ratio from 4.5 to 3.7 (18% reduction), and the C_20:1_/C_20:0_ ratio from 1.3 to 0.8 (39% reduction) (Fig. 5B). Conversely, the C_16:1_/C_16:0_ ratio had a slight increase from 0.036 to 0.044 (22% increase) (Fig. 5B). We also performed a lipidomic analysis on cells grown with or without half the myriocin MIC for 48 h. As expected, the most abundant ceramide species, Cer(d18:1/24:0(2OH)) and Cer(d20:0/24:0), were reduced by 52% and 62%, respectively (Fig. 5C). As a control of a lipid from an unrelated class, the level of the most abundant DG, DG(16:0/18:2/0:0), increased by 51% (Fig. 5C).

**FIG 6:**
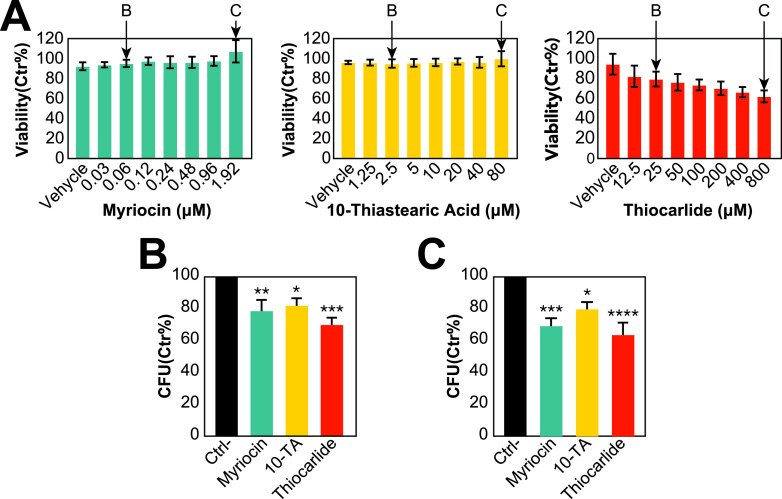
Toxicity and efficacy of lipid metabolism inhibitors against intracellular infection of AMJ2 alveolar macrophages with Histoplasma capsulatum. (A) Viability of AMJ2 alveolar macrophages, measured by MTT (3-[4,5-dimethylthiazol-2-yl]-2,5-diphenyltetrazolium bromide) assay, after exposed to different concentrations of myriocin, 10-thiastearic acid, and thiocarlide. (B and C) Intracellular killing of H. capsulatum by AMJ2 alveolar macrophages treated with the low and high concentrations of myriocin, 10-thiastearic acid (10-TA), and thiocarlide. The concentrations are highlighted in panel A. Bars represent the combination of 4 independent experiments with 3 replicates each (*n* = 12). *, *P* ≤ 0.05; **, *P* ≤ 0.01; and ***, *P* ≤ 0.001 by one-way ANOVA followed by Dunnett’s test comparing the control and treatments.

To further test the fatty acid desaturation and sphingolipid pathway as anti-H. capsulatum drug targets, we tested the ability of these inhibitors to reduce intracellular infection. We chose alveolar macrophages, as they are the primary cells targeted by H. capsulatum in infection (30). We performed a toxicity assay with concentrations up to 64-fold higher than the MICs. Myriocin 10-thiastearic acid did not affect the viability of AMJ2 alveolar macrophages, while thiocarlide had a small effect, reducing the viability of the cells by approximately 30% on the highest tested concentrations (Fig. 6A). We tested a low (2× MIC; 0.06 μM myriocin, 2.5 μM 10-thiastearic acid, and 25 μM thiocarlide) and a high (2 μM myriocin, 80 μM 10-thiastearic acid, and 50 μM thiocarlide) concentration to determine the ability of the compounds to reduce intracellular infection. In low concentrations, myriocin, 10-thiastearic acid, and thiocarlide reduced intracellular H. capsulatum load by 21.4%, 19.2%, and 30.1%, respectively (Fig. 6B), while the higher concentration further reduced this by 32.0%, 21.4%, and 37.4% (Fig. 6C). These results validate the fatty acid desaturation and sphingolipid pathways as a potential target for developing anti-H. capsulatum drugs and that thiocarlide can reduce intracellular fungal load in alveolar macrophages.

## DISCUSSION

Here, we developed a global map of the H. capsulatum lipid metabolism by incorporating genomic, proteomic, and lipidomic information, including relative abundances of proteins and lipids. We found accordance of the protein abundance levels with their functions in lipid metabolism. For instance, the proteins involved in the synthesis of the major lipid components, the fatty acids and sterols, were enriched among the high- and moderate-abundance proteins, whereas the proteins related to the synthesis of specific lipid head groups were enriched in the low- to very-low-abundance proteins. This might be a consequence of cell resource optimization, as protein synthesis is one of the most energetically expensive tasks in cells (31). Our lipid map also showed that TG, PC, and PE are the most diverse classes of lipids, which is in agreement with the fact that they are the most abundant ones (32). Our results show that the diversity of TG, PC, and PE species could be due to fatty acid remodeling, as these lipids are products of the LPT1 acyltransferase. LPT1 transfers acyl groups to a variety of fatty acyl chains, including odd-numbered carbon chains (C_15_ and C_17_) and long-chain (≥C_18_) fatty acids. Our experiments with LPT1 also helped to curate the lipid map, since the S. cerevisiae homolog has a different specificity for substrates, having a major impact on producing phospholipids with short fatty acyl chains. In S. cerevisiae, the substrates of LPT1 have been shown *in vitro* using radioactive precursors to acylated LPC, LPG, LPA, LPE, LPI, and LPS (27). The acylation of LPA, a central precursor for all glycerolipids and glycerophospholipids, suggests that the LPT1 impact is indirect. Therefore, further investigation will be needed to determine if this enzyme can directly acylate DG into TG. We also curated the fatty acid desaturation pathway by expressing H. capsulatum desaturase candidate genes in S. cerevisiae. We validated that Ole1 has delta-9 desaturase activity. In addition, we show that FAD2 is indeed a bifunctional delta-12/delta-15 desaturase. In fungi, delta-15 and bifunctional delta-12/delta-15 desaturases evolved from delta-12 desaturases in multiple independent gene duplications, which complicates the assignment of their specificity based on their sequence (28). This scenario is a little different in H. capsulatum, as H. capsulatum FAD2 evolved to have both activities within a single gene copy rather than duplicating the gene.

We identified the fatty acid desaturation and the sphingolipid pathways as divergent points in the lipid metabolism pathways of H. capsulatum versus S. cerevisiae and humans. We showed that both fatty acid and sphingolipid pathways are potential targets for developing anti-histoplasmosis drugs. Thiocarlide (also known as isoxyl) is a potent inhibitor (nanomolar range) of Mycobacterium tuberculosis delta-9 stearoyl desaturase and was used as a second line of antituberculosis drugs in the 1960s (33, 34). However, despite its low toxicity, thiocarlide was not as effective during clinical trials, so it is no longer used to treat patients (34–36). One possible explanation for the clinical trial failure is the poor solubility of the compound in water. Since then, other drug delivery vehicles have been tested with promising results *in vitro* (37). Delta-12 oleate desaturase has been validated as a drug target in the protozoan parasite Trypanosoma cruzi (38). Indeed, thiocarlide inhibited T. cruzi growth on a low micromolar range and reduced the levels of unsaturated fatty acids by approximately 30% (at 10 μM concentration) (39). These numbers are similar to the MIC and reduction in unsaturated fatty acid level for H. capsulatum (Fig. 5A and B). The small changes in unsaturated fatty acid levels were associated with a drastic reduction in cell viability, which suggests that maintenance of homeostasis in membrane fluidity is crucial for life. Moreover, thiocarlide was able to reduce intracellular infection with H. capsulatum. The small reduction in intracellular infection might be due to limited diffusion through the host cell membrane, which is observed in current antifungal drugs such as voriconazole, and increased concentrations are required to kill intracellular fungi (40). Such desaturase inhibitors should also be tested in other pathogenic fungi, as many of them produce polyunsaturated fatty acids (41, 42).

Our data also showed that 10-thiastearic acid might target a pathway other than fatty acid desaturation. This is expected to some extent, since 10-thiastearic acid is a fatty acid analog; therefore, it is more likely to inhibit other processes of fatty acid metabolism. However, this type of molecule should still be considered a drug candidate due to its antifungal activity and potential low toxicity to humans. Our data showed no toxicity in AMJ2 alveolar macrophages with up to a 64-fold MIC of 10-thiastearic acid. In addition, the 10-thiastearic acid analog tetradecylthioacetic acid has a low toxicity in humans up to 1 g/day (43). Engineered delivery methods, such as liposomes, and structural modifications, such as esterification, can further improve the bioactive compound to reach the intracellular milieu. Therefore, 10-thiastearate might still be useful for treating histoplasmosis, but further development still needs to be performed to test their efficacy.

We also performed a proof of concept that sphingolipids are promising targets for anti-histoplasma drug development by inhibiting the first step of the sphingolipid pathway with myriocin. Myriocin kills Candida albicans, C. auris, and Aspergillus fumigatus in nanomolar to micromolar concentrations (17, 44, 45). H. capsulatum is more sensitive to myriocin, as its MIC was 30 nM (Fig. 5B). Myriocin was also able to reduce the intracellular fungal load. Unfortunately, myriocin has immunosuppressant activity (46, 47), but this has been explored for simultaneously killing the fungus and reducing the pathogenic inflammation of the lungs in cystic fibrosis mice infected with A. fumigatus (48). The fact the *de novo* sphingolipid synthesis is essential for H. capsulatum opens the opportunity to explore other inhibitors of serine palmitoyltransferase or other enzymes in the pathway. Indeed, other enzymes of the fungal sphingolipid pathway have been validated as drug targets. In C. neoformans, acylhydrazones have been shown to be potent inhibitors of glucosylceramide synthesis (nanomolar range) and excellent antifungal drug candidates (13, 49). Sphingolipids have also been shown to be a mechanism of azole resistance in C. albicans (50) and, at the same time, excellent targets for synergistic drugs across multiple species (51).

In conclusion, we built a detailed map of H. capsulatum lipid metabolism based on omics data. Our data identified defined regions of the H. capsulatum lipid metabolic pathway that can be targeted for drug development. The lipid metabolic map is a valuable resource to the community, and its use can help in the discovery of other functions of lipids in fungal physiology and pathogenesis.

### Limitations of study.

One limitation of this study is that H. capsulatum is a fungus with few genetic engineering tools, which makes the execution of gene knockout or knockdown experiments highly challenging. Therefore, we utilized the gene complementation system in S. cerevisiae to study the specificity of genes from lipid metabolism. We found dozens of H. capsulatum LPT1 products and determined the double-bond positions catalyzed by FAD2, but the complementation system can generate false negatives due to the absence of specific substrates in S. cerevisiae. The different lifestyles between H. capsulatum and S. cerevisiae limit some of the comparisons between the lipid composition of these two organisms. Another limitation of our study is that the H. capsulatum lipidomic analysis was performed in a single growth condition, which might not fully recapitulate the *in vivo* environment during infection. We also have previously shown that different growth conditions have an impact on the composition of extracellular vesicles, including variations in lipid profiles (52). Therefore, the analysis of different growth conditions and forms of the fungus might lead to different lipid profiles and even the identification of additional lipid species. The current work was performed in a single H. capsulatum strain (G217B). Strain-dependent variations regarding lipid and protein profiles are expected among different fungal strains of the same species, which needs to be further studied in future work.

## MATERIALS AND METHODS

### Cells.

Histoplasma capsulatum G217B strain and murine alveolar macrophage AMJ2 were purchased from the American Type and Culture Collection (ATCC; Manassas, VA). S. cerevisiae deficient in the LPT1 gene (YOR175C knockout [KanMX], strain 12431) and wild-type strains (BY4742; *mat*α *his3Δ1 leu2Δ0 lys2Δ0 ura3Δ0*) were purchased from Dharmacon (Lafayette, CO).

### Saccharomyces cerevisiae transformation.

Strains were maintained on YPD (2% glucose, 10 g/liter peptone, 10 g/liter yeast extract) or YNB with amino acid supplements (2% glucose, 1.7 g/liter YNB salts without ammonium sulfate or amino acids [Difco], 5 g/liter ammonium sulfate [Fisher], with or without 50 mg/liter leucine, lysine, histidine [LLH] and uracil). The strains were transformed by a lithium-acetate heat shock method (53) with a plasmid containing the H. capsulatum acyltransferase LPT1 (UniProt ID C0NZS2) or desaturases Ole1 (UniProt ID C0NLE5), FAD2 (UniProt ID C0NKL1), HCBG_08514 (UniProt ID C0NZD4), and HCBG_05092 (UniProt ID C0NPL2) genes and S. cerevisiae Ole1 (UniProt ID P21147) that were codon optimized, synthesized, and cloned into the Gal-inducible plasmid pESC-URA by GenScript (Piscataway, NJ). Briefly, 500 μl of cells grown in YPD for 16 h was spun down and the supernatant removed. Added to the BY4742 and 12431 cells were 5 μg of herring sperm DNA (5 μl of 10 mg/ml stock), 400 ng of GenScript plasmid DNA, 500 μl of PLATE buffer (40% polyethylene glycol 3350, 0.1 M lithium acetate, 10 mM Tris-HCl [pH 8.0], 1 mM EDTA), and 57 μl dimethyl sulfoxide. Cells were incubated at room temperature for 15 min, followed by 15 min at 42°C. Cells were collected by brief centrifugation of 5 s at 8,000 × *g*, and supernatant was removed, resuspended in phosphate-buffered saline (PBS), and plated on YNB-2% glucose-LLH 2% agar plates without uracil. Resulting colonies were screened for plasmid presence by PCR of a portion of the pESC-URA-lpt1 plasmid (forward primer, 5′-TTGGAAACAGCTCCAAATCC-3′; reverse primer, 5′-CCCAAAACCTTCTCAAGCAA-3′; ordered from ThermoFisher Oligos) and preserved as glycerol stocks.

### S. cerevisiae plasmid expression assays.

For the Hc_LPT1 and desaturase expression assays, cells were precultured overnight in YNB 2% glucose LLH with or without uracil to accommodate the nonplasmid strain auxotrophy as a negative control. For biomass collection during induction, which often slows or prevents cell division, cells were inoculated to an optical density of 0.5 into YNB-2% galactose LLH with or without uracil and collected after induction of plasmid expression for 17 h. LPT1 cultures were also analyzed after 17 h of growth in glucose LLH with or without uracil (uninduced). Cultures were collected by spinning 25 ml of culture in a tabletop centrifuge (VWR symphony 4417 R) at 2,000 × *g* for 2 min with a swinging-bucket rotor fitting 50-ml conical tubes. Pellets were collected into preweighted tubes and washed 2× with TBS before flash freezing in liquid nitrogen. All samples were made in 4 replicates as input for downstream omics analysis.

### Histoplasma capsulatum cell culture.

Yeasts of H. capsulatum G217B were grown in Ham’s F12 medium at 37°C under constant shaking and used 3 days after its inoculation, as previously described (54). Cells were harvested by centrifuging at 2,000 × *g* for 2 min and washed twice with PBS. All of the sample preparation and analysis were done in 4 replicates and in a randomized order to ensure biological significance and prevent batch effects.

### Sample extraction and fractionation on Silica 60 column.

Samples were extracted using two different approaches. For global proteomics and lipidomics analyses, samples were submitted to Metabolite, Protein and Lipid Extraction (MPLEx) as previously described (23). For the fractionation study, cells were extracted twice with chloroform-methanol (2:1, vol/vol) and twice with chloroform-methanol-water (1:2:0.8, vol/vol/vol) as described elsewhere (19). Extracted lipids were fractionated into neutral lipids, fatty acids, and phospholipid fractions using Silica 60 columns (55) and dried in a vacuum centrifuge.

### Proteomic analysis.

Extracted proteins were digested and analyzed by LC-MS/MS as described previously (56). Data were analyzed with MaxQuant software (v.1.5.5.1) by searching against the H. capsulatum G186AR (downloaded 15 August 2016) and S. cerevisiae S288c (downloaded 11 January 2018) sequences from UniProt Knowlegdgebase, considering protein N-terminal acetylation and oxidation of methionine as variable modifications and cysteine carbamidomethylation as a fixed modification. The remaining parameters were set as the software default. Protein abundances were estimated by the intensity-based absolute quantification (iBAQ) method (57). The intensities were normalized by the total iBAQ sum of each sample and expressed as the relative number of protein copies (percent from total). Proteins were classified according to their abundance, converting number of copies described by Beck et al. (24) to the relative number of protein copies (%). Function enrichment analysis was performed as described previously (58).

### Lipid analysis by LC-MS/MS.

Total lipid extracts and phospholipid fraction from the Silica 60 column were resuspended in methanol and subjected to LC-MS/MS analysis as previously described (59). To assess the technical variability, we spiked in the SPLASH Lipidomix standard (Avanti Polar), which contains a mix of isotopically labeled lipids. Lipid species were identified using LIQUID, which matches spectra against a comprehensive database of lipid species, including all the species contained in LipidMaps in addition to several other classes of lipids recently described in the literature (60). Identified species were manually inspected for validation based on isotopic distribution and head group and fatty acyl fragments. The features of the identified lipid species were extracted and aligned using MZmine (61). For comparative purposes, lipids were considered significantly different with *P* ≤ 0.05 by *t* test considering equal variance and unpaired samples. The distribution of fatty acyl groups was done by counting individual fatty acyl groups and was considered significantly different with a *P* value of ≤0.05 by Fisher’s exact test.

### Fatty acid and sterol analyses.

Fatty acids were methylated with anhydrous methanolic HCl (1.2 N) for 1 h at 100°C and extracted by adding equal volumes of water and hexane. Sterols were treated with 30 mg/ml methoxyamine in pyridine for 90 min at 37°C with shaking and derivatized with N-methyl-N-(trimethylsilyl)trifluoroacetamide (MSTFA) (Sigma-Aldrich) with 1% trimethylchlorosilane (TMCS) (Sigma-Aldrich) at 37°C with shaking for 30 min (62). Derivatized FAMEs and sterols were then analyzed in an Agilent GC 7890A using an HP-5MS column (30 m by 0.25 mm by 0.25 μm; Agilent Technologies, Santa Clara, CA) coupled with a single-quadrupole MSD 5975C (Agilent Technologies). Samples were injected (splitless) into the port set at 250°C with an initial oven temperature of 60°C. After 1 min, the temperature was increased to 325°C at a rate of 10°C/min and finished with a 5-min hold at 325°C. The data files were calibrated with external calibration of FAME standards (C8-28; Sigma-Aldrich) and deconvoluted with Metabolite Detector as stated previously (63). Molecules were identified by library matching against the FiehnLib (64) with additional in-house entries, the Wiley Fatty Acids Methyl Esters Mass Spectral Database, and the NIST17 GC-MS spectral library.

### Evaluation of lipid biosynthesis inhibitors on H. capsulatum axenic and intracellular growth.

H. capsulatum yeasts were washed with PBS, suspended in Ham’s F12, and loaded into plates with serially diluted compounds at a final cellular density of 2.5 × 10^4^ cells/ml in 4 replicates. Cells were grown at 37°C under constant shaking for 7 days, and minimal drug concentration with no visual growth was considered the MIC. To evaluate the effect of lipid synthesis inhibition of H. capsulatum, triplicates of 10^6^ cells/ml were incubated in Ham’s F12 at 37°C with shaking at 250 rpm for 48 h with half the MIC of myriocin, 10-thiasteric acid, and thiocarlide. Cells were washed with PBS and pellets were frozen for further lipid extraction and subsequent fatty acid and lipidomics analyses. To assess the ability of the compounds to kill intracellular fungi, murine alveolar macrophage cell line AMJ2 was plated onto 96-well plates and incubated overnight at 37°C with 5% CO_2_. The macrophages were challenged with H. capsulatum (ratio of 2 yeasts per macrophage cell) in the presence or absence of myriocin, 10-thiasteric acid, and thiocarlide for 48 h (see the figure legends for concentrations). Macrophages were lysed with distilled water and the yeast suspensions were diluted and plated onto brain heart infusion agar supplemented with 5% sheep’s blood. Plates were incubated at 37°C and CFU were counted after growth. To address cytotoxicity of the inhibitors on alveolar macrophages, AMJ2 cells were plated (10^5^ cells/well) in 96-well plates and incubated overnight at 37°C and 5% CO_2_. Cells were washed with PBS and incubated with serially diluted inhibitors. Solvent controls were made with the same solvent amount present in the highest concentration for each inhibitor. Plates were incubated for 24 h at 37°C and 5% CO_2_. MTT (3-[4,5-dimethylthiazol-2-yl]-2,5-diphenyltetrazolium bromide) was added to each well (50 μg/well) and incubated for 4 h at 37°C and 5% CO_2_. After supernatant removal, the formazan crystals were solubilized with isopropanol and read with a plate reader at 570 nm. All values were normalized using the negative control (absence of drug or solvent).

### Construction of the H. capsulatum lipid metabolic map.

We built a preliminary G217B lipid map with a tool name VANTED v2.6.5 (65), using the S. cerevisiae model (25) as a starting point. Enzyme homologs were identified by performing BLAST searches with S. cerevisiae and C. neoformans, two of the best-characterized fungi in terms of lipid metabolism (25, 66). We also based the map on the information available on H. capsulatum from the lipid literature and lipids identified in the lipidomics analysis. The map was integrated by adding the abundance level of lipids using their relative mass spectrometry intensities within the same lipid class.

### Lipidomics and proteomics visualization tool.

We developed a software tool based on the lipid metabolic map to automatically build a figure representing lipidomics and proteomics data. Colorblind-friendly palettes were applied to the relative abundance values and mapped to a static image displaying lipid metabolism pathways. The script was written in Perl using the GD graphics package.

### Data availability.

Proteomics data were deposited into the Pride repository (www.ebi.ac.uk/pride) under accession number PXD017734. The code for the lipidomics visualization tool is available under the BSD 2-Clause License at GitHub at https://github.com/wichne/LipidomicsMapTools.
